# Radical Prostatectomy Following Holmium Laser Enucleation of the Prostate (HoLEP): A Systematic Review of Perioperative, Oncological, and Functional Outcomes

**DOI:** 10.3390/cancers17223685

**Published:** 2025-11-18

**Authors:** Stamatios Katsimperis, Lazaros Tzelves, Titos Markopoulos, Themistoklis Bellos, Konstantinos Douroumis, Nikolaos Kostakopoulos, Andreas Skolarikos

**Affiliations:** 1 Second Department of Urology, National and Kapodistrian University of Athens, Sismanogleio Hospital, 15126 Athens, Greece; 2 Department of Urology, Red Cross General Hospital of Athens, 11526 Athens, Greece; 3 First Department of Urology, National and Kapodistrian University of Athens, 11527 Athens, Greece; 4 First Department of Urology, Metropolitan General Hospital, 15562 Athens, Greece

**Keywords:** radical prostatectomy, holmium laser enucleation of the prostate (HoLEP), prostate cancer, benign prostatic hyperplasia (BPH), robot-assisted radical prostatectomy (RARP), perioperative outcomes, functional outcomes, oncological outcomes, surgical feasibility, urinary continence recovery

## Abstract

**Background**: The widespread adoption of holmium laser enucleation of the prostate (HoLEP) has led to a growing number of men subsequently diagnosed with localized prostate cancer requiring radical prostatectomy (RP). However, anatomical alterations after HoLEP may increase surgical complexity and affect outcomes. This systematic review aimed to synthesize current evidence on perioperative, oncological, and functional outcomes of RP following HoLEP. **Methods**: A systematic literature search was conducted in PubMed, CENTRAL, and ClinicalTrials.gov through to September 2025 in accordance with PRISMA 2020 guidelines (PROSPERO CRD420251134483). Eligible studies included patients undergoing RP after HoLEP with reported perioperative, oncologic, or functional data. Methodological quality was assessed using the ROBINS-I tool, and results were synthesized narratively. **Results**: Eight retrospective studies comprising 202 patients were included. RP after HoLEP was technically feasible across open, laparoscopic, and robotic approaches. Operative time and the need for bladder-neck reconstruction were increased, reflecting post-enucleation fibrosis, but major complication rates (<5%) and blood loss were comparable to primary RP. Oncological outcomes were preserved, with positive surgical margin rates of 6–20% and biochemical recurrence rates of 7–15%, similar to those of primary RP. Functional recovery, particularly urinary continence, was slower initially but generally equivalent at 12 months. Erectile function outcomes were variable but satisfactory when nerve-sparing was feasible. **Conclusions**: Radical prostatectomy after HoLEP is a technically demanding yet safe procedure that achieves oncologic and long-term functional outcomes comparable to primary prostatectomy. Prior HoLEP should not preclude curative surgical management of prostate cancer, provided the operation is performed by experienced surgeons in high-volume centers.

## Simple Summary

Holmium laser enucleation of the prostate (HoLEP) has become a gold-standard treatment for benign prostatic hyperplasia, and an increasing number of these patients are later diagnosed with prostate cancer requiring radical prostatectomy. However, prior HoLEP alters prostatic anatomy, raising concerns about surgical safety and functional recovery. This systematic review summarizes the available evidence on radical prostatectomy after HoLEP, evaluating perioperative, oncological, and functional outcomes across eight studies involving 202 patients. The findings demonstrate that the procedure is technically more demanding and often associated with longer operative times and the need for bladder-neck reconstruction, but major complications remain rare. Oncological control and long-term functional outcomes are comparable to primary prostatectomy, although early continence recovery may be delayed. Overall, radical prostatectomy after HoLEP is feasible, safe, and effective when performed by experienced surgeons in specialized centers.

## 1. Introduction

Holmium laser enucleation of the prostate (HoLEP) has emerged as a highly effective, evidence-based surgical option for benign prostatic hyperplasia (BPH), offering durable symptom relief and favorable safety outcomes [1,2,3]. Its precise anatomical dissection along the surgical capsule allows complete removal of the adenomatous tissue while preserving periprostatic structures. As HoLEP has become increasingly widespread, a growing number of men with a prior history of the procedure are subsequently being diagnosed with localized prostate cancer and considered for radical prostatectomy [4,5]. Given the aging population and expanding indications for HoLEP, this scenario is expected to become increasingly common in contemporary urologic practice.

Radical prostatectomy (RP), performed through open, laparoscopic, or robot-assisted approaches, remains a cornerstone in the management of clinically localized prostate cancer [6]. However, prior transurethral or endoscopic surgery—such as transurethral resection of the prostate (TURP) or HoLEP—can alter key anatomical landmarks, particularly at the bladder neck and prostatic apex [7,8]. These changes may complicate subsequent dissection, obscure surgical planes, and raise concerns regarding perioperative safety, oncological adequacy, and functional recovery [9,10]. While the impact of previous TURP on RP outcomes is well documented, the specific effects of prior HoLEP remain less clearly defined, partly because HoLEP creates a distinct and more anatomically precise enucleation cavity.

Historically, radical prostatectomy performed after TURP was associated with increased blood loss, prolonged operative time, and inferior postoperative functional outcomes [11,12,13]. HoLEP, however, differs fundamentally from TURP by achieving a controlled anatomical enucleation within the true capsular plane, theoretically minimizing collateral damage and preserving the integrity of periprostatic tissues. Whether these anatomical advantages translate into improved outcomes for patients undergoing RP after HoLEP remains unclear. Given the rising number of post-HoLEP patients requiring definitive cancer treatment, understanding surgical feasibility and outcome expectations has become increasingly important for clinical decision-making.

The aim of this systematic review was therefore to synthesize all available evidence on perioperative, oncological, and functional outcomes of radical prostatectomy in patients with a history of HoLEP. By consolidating existing data, this review seeks to clarify the feasibility, safety, and long-term efficacy of RP in this increasingly relevant clinical scenario. Such evidence may help guide preoperative counseling, refine surgical planning, and improve patient selection as HoLEP continues to gain prominence worldwide.

## 2. Methods

### 2.1. Search Strategy and Selection Criteria

**Figure 1:**
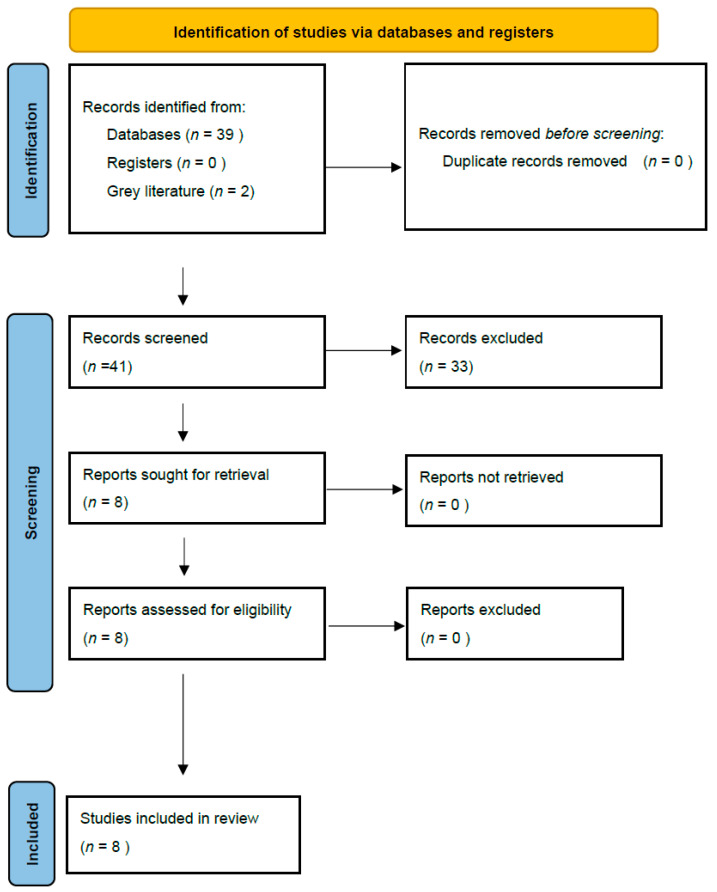
PRISMA flow diagram summarizing the identification, screening, eligibility, and inclusion of studies evaluating radical prostatectomy after HoLEP.

This systematic review was conducted in accordance with the PRISMA guidelines [14]. A comprehensive literature search was performed using PubMed, the Cochrane Central Register of Controlled Trials (CENTRAL), and ClinicalTrials.gov from database inception through September 2025 (**Figure 1**). The protocol was registered to PROSPERO (CRD420251134483).

The following search terms were applied:

(radical prostatectomy) AND (HoLEP OR holmium laser enucleation).

Reference lists of included articles were manually screened to identify additional relevant studies.

Studies were included if they met the following criteria: (1) reported outcomes of radical prostatectomy performed after prior HoLEP for BPH; (2) provided data on perioperative, oncological, or functional outcomes; and (3) were published in English as full-text articles or abstracts with extractable data. Conference abstracts were included only if they provided sufficient extractable outcome data. These criteria were selected to ensure that the review focused exclusively on radical prostatectomy performed after clearly documented HoLEP, as outcomes from mixed or unspecified prior procedures may not be comparable. Exclusion criteria were (1) studies that did not specifically identify HoLEP as the prior procedure, (2) those combining HoLEP with other BPH surgeries without stratified results, (3) case reports, review articles, or non-English publications without accessible translation. Studies combining HoLEP with other BPH procedures without stratified reporting were excluded to avoid misattribution of outcomes.

To ensure completeness, gray literature was screened for relevant conference abstracts and unpublished studies.

### 2.2. Data Extraction and Bias Assessment

Two reviewers (S.K., L.T.) independently screened titles and abstracts, followed by full-text evaluation of eligible studies. Extracted data included study design, patient demographics, surgical approach, interval between HoLEP and RP, operative time, estimated blood loss, complications, bladder-neck reconstruction, positive surgical margins (PSM), biochemical recurrence (BCR), continence, and erectile function outcomes. Discrepancies were resolved by consensus.

Given the non-randomized design of all eligible studies, methodological quality was evaluated using the Risk of Bias in Non-Randomized Studies of Interventions (ROBINS-I) tool [15]. Each study was independently assessed across seven domains: bias due to confounding, selection of participants, classification of interventions, deviations from intended interventions, missing data, measurement of outcomes, and selection of the reported result. Two reviewers (S.K., L.T.) performed the assessment independently, resolving discrepancies by consensus. Judgments were classified as low, moderate, serious, critical, or no information according to the ROBINS-I guidance.

**Figure 2:**
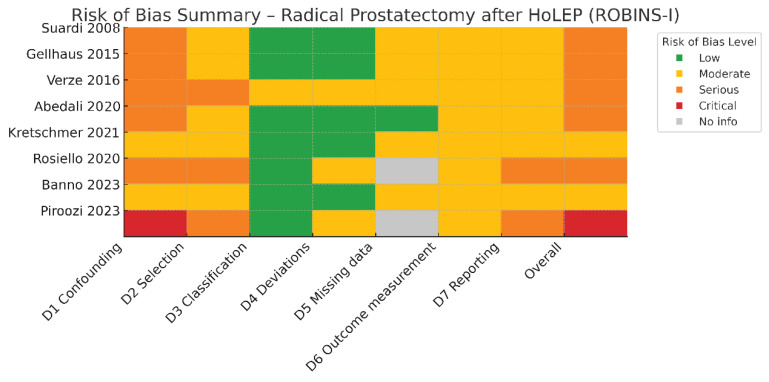
Risk of bias assessment of the included studies according to the ROBINS-I tool [16,17,18,19,20,21,22,23].

The overall risk of bias for each study corresponded to the highest level of bias identified in any domain. Results were summarized in a traffic-light figure (**Figure 2**) created using the standard ROBINS-I color scale (green = low, yellow = moderate, orange = serious, red = critical, gray = no information). This figure provides a visual overview of domain-specific judgments for each study, facilitating comparison of methodological rigor across the evidence base. The risk-of-bias assessment demonstrated moderate to serious overall bias across most included studies, reflecting their retrospective, non-randomized design and small sample sizes. Confounding bias was the predominant limitation. Selection bias was moderate to serious in most studies because patients undergoing RP after HoLEP were typically treated in tertiary or high-volume centers. Classification bias was uniformly low, since prior HoLEP status was clearly documented. Bias due to deviations from intended interventions was low to moderate, and missing-data bias was moderate owing to incomplete follow-up in several reports. Outcome measurement bias was generally moderate: continence and erectile function were frequently based on patient-reported outcomes, but assessor blinding was absent. Reporting bias was moderate in full-text publications but serious in abstract-only studies, where methodological details were sparse. A detailed summary of domain-specific judgments is presented in **Figure 2**.

Given the heterogeneity in study design and outcome reporting, a meta-analysis was not feasible. Instead, results were synthesized narratively and categorized into perioperative, oncological, and functional outcomes.

## 3. Results

### 3.1. Study Characteristics

Eight studies met the inclusion criteria, comprising six full-text retrospective series and two conference abstracts, with a total of 202 patients who underwent radical prostatectomy after previous HoLEP (**Table 1**) [16,17,18,19,20,21,22,23]. All included studies were retrospective observational series with small sample sizes, reflecting the uncommon nature of radical prostatectomy performed after prior HoLEP. Most procedures were performed with a robot-assisted approach, while a smaller proportion were conducted laparoscopically or via open surgery. Median follow-up ranged from 12 to 60 months across studies, and the majority of cohorts originated from high-volume academic centers.

### 3.2. Perioperative Outcomes

All studies consistently demonstrated that radical prostatectomy following HoLEP is feasible and safe, though technically more demanding than primary surgery. Operative times were generally longer and bladder-neck reconstruction was required more frequently in post-HoLEP patients, yet perioperative morbidity remained comparable. Gellhaus et al. reported significantly prolonged operative time (216.6 vs. 164.6 min, *p* = 0.005) and higher estimated blood loss (209.1 vs. 130.7 mL, *p* = 0.012) compared with controls without prior HoLEP, findings echoed by Abedali et al., who observed similar trends [17,18]. Despite the technical complexity, major complications (Clavien–Dindo grade ≥ III) were rare, occurring in fewer than 3% of patients.

The need for bladder-neck reconstruction was more common in the HoLEP group, likely reflecting anatomical alterations such as scarring and wider bladder-neck openings. However, these reconstructions did not result in longer catheterization or hospitalization times. Banno et al. also observed a modest increase in operative duration but found no significant differences in blood loss or complication rates [21]. Verze et al. confirmed prolonged console time but reported stable perioperative morbidity [20]. Conference data by Rosiello et al. supported these observations, noting slightly higher operative times and blood loss but no increase in major complications among post-HoLEP patients [22]. Collectively, these findings indicate that prior HoLEP modestly increases surgical difficulty but does not compromise the safety profile or overall feasibility of radical prostatectomy.

### 3.3. Oncological Outcomes

Oncological efficacy was preserved across all available studies. Positive surgical margin (PSM) rates ranged between 6% and 20%, comparable to those reported for primary radical prostatectomy. Kretschmer et al. found no significant difference in PSM between patients with and without prior HoLEP (14.0% vs. 18.8%, *p* = 0.06), while Abedali et al. observed improvement over time, with no positive pT2 margins reported after 2010 [18,19]. BCR was reported heterogeneously across studies. Three studies provided time-dependent biochemical recurrence-free survival using Kaplan–Meier analysis, all showing no significant difference from controls [19,20,21]. Other studies (Abedali, Gellhaus, Rosiello) reported only crude BCR proportions at last follow-up (7–14%) [17,18,22]. Suardi et al. and Piroozi et al. did not report BCR outcomes [16,23]. In the series by Banno et al., PSM and seminal vesicle invasion were independent predictors of recurrence, whereas prior HoLEP was not [21].

No cancer-specific deaths were reported in any of the included studies. Similarly, Rosiello et al. documented only one biochemical recurrence among seven patients with prior HoLEP, comparable to five recurrences among 19 controls [22]. These findings collectively affirm that radical prostatectomy after HoLEP achieves oncological outcomes equivalent to those of primary procedures when performed by experienced surgeons.

### 3.4. Functional Outcomes

Functional recovery after radical prostatectomy in patients with previous HoLEP showed greater variability, particularly regarding urinary continence. Early postoperative continence recovery tended to be slower, although long-term outcomes were ultimately similar. Gellhaus et al. reported that 27% of patients achieved strict continence (0 pads/day) compared with 64% of controls (*p* = 0.071), with a median recovery time of 12 vs. 6 months (*p* = 0.041) [17]. Abedali et al. found comparable patterns, with 22% vs. 74% achieving continence at 12 months (*p* < 0.001) and longer recovery times (20 vs. 6 months, *p* = 0.007) [18]. In contrast, Kretschmer et al. and Banno et al. demonstrated that continence rates eventually approximated those of primary radical prostatectomy, and multivariate analyses did not identify prior HoLEP as an independent predictor of persistent incontinence [19,21].

Evidence from conference abstracts reinforced these trends. Rosiello et al. reported continence rates of 14.2% at one month and 71.4% at three months, comparable to 15.8% and 57.9% among controls, respectively [22]. Piroozi et al. compared radical prostatectomy and radiotherapy after HoLEP, observing satisfactory continence rates at 12 months in the surgical group, underscoring the functional viability of radical prostatectomy in this context [23].

Data on erectile function were limited due to the older patient population and the infrequent application of nerve-sparing techniques. Early studies suggested compromised erectile recovery, likely secondary to apical fibrosis, whereas more recent robotic series demonstrated that when nerve-sparing is technically feasible, potency recovery rates can approach those achieved in primary radical prostatectomy.

Definitions of continence and potency varied across studies and are summarized in the final column of **Table 1**. Most studies defined continence as complete pad freedom (0 pads/day), although two accepted the use of up to one safety pad per day. Potency was variably assessed using Sexual Health Inventory for MenSHIM scores, patient-reported erectile function, or was not defined at all. This heterogeneity likely contributed to the variability in reported recovery rates and limits direct comparison across studies.

Perioperative, oncological and functional outcomes across studies are summarized in **Table 2**.

## 4. Discussion

This systematic review demonstrates that radical prostatectomy following HoLEP is technically feasible and oncologically safe when performed by experienced surgeons. Across the available literature, the procedure is consistently associated with greater technical difficulty, longer operative times, and a higher likelihood of bladder-neck reconstruction, yet perioperative safety, oncologic adequacy, and long-term functional outcomes remain comparable to those achieved in primary radical prostatectomy.

HoLEP fundamentally alters intraprostatic anatomy by dissecting along the surgical capsule, creating a thin residual prostatic rim and a widely open bladder neck [24]. As a result, subsequent radical prostatectomy is performed in a field often characterized by fibrosis and disrupted tissue planes, making anatomical identification and dissection more challenging. These anatomical changes explain the consistently observed increase in operative time and the need for bladder-neck reconstruction across studies. Despite these challenges, the incidence of major complications remains low, and estimated blood loss and transfusion rates are mildly higher to those in patients without prior HoLEP but not clinically significant. The evolution of robotic-assisted techniques has further mitigated intraoperative difficulty by providing enhanced visualization and instrument precision, allowing safer dissection within fibrotic planes and minimizing morbidity.

Importantly, oncological safety appears to be preserved following HoLEP. Positive surgical margin and biochemical recurrence rates are comparable to those of patients undergoing primary radical prostatectomy, and prior HoLEP has not been identified as an independent predictor of oncological failure. These findings indicate that adequate oncologic resection can be achieved despite altered anatomy. The preservation of the prostatic capsule after HoLEP, when combined with meticulous apical dissection and surgical expertise, likely contributes to these favorable oncologic results.

Functional recovery, particularly urinary continence, represents the most challenging aspect of post-HoLEP radical prostatectomy. Several studies have reported slower early continence recovery, likely due to apical fibrosis and distortion of the sphincteric complex after enucleation. Nevertheless, most series show that continence rates converge with those of primary radical prostatectomy over time, underscoring that delayed recovery does not necessarily translate into poorer long-term outcomes. Technical refinements, including careful reconstruction of the posterior musculofascial plate, meticulous preservation of the sphincter mechanism, and early pelvic floor training, may help mitigate these functional drawbacks.

Data on erectile function remain limited, reflecting both the older age of most patients and the relatively low frequency of nerve-sparing procedures in this setting. However, recent robotic series have demonstrated that when nerve-sparing is anatomically feasible, erectile function recovery can approach rates observed in primary radical prostatectomy [21]. These findings emphasize the importance of individualized surgical planning and intraoperative judgment to optimize both oncological and functional outcomes.

Comparison with historical data from patients undergoing radical prostatectomy after TURP or open simple prostatectomy provides additional context [25,26]. TURP often disrupts the prostatic capsule irregularly, causing unpredictable scarring and more complex dissection during subsequent prostatectomy. In contrast, HoLEP follows a controlled anatomical plane along the true capsule, producing less distortion of periprostatic anatomy. This anatomical advantage likely explains why perioperative and long-term functional outcomes after HoLEP are superior to those observed after TURP or open procedures. Consequently, the growing adoption of HoLEP for benign prostatic hyperplasia should not be viewed as a barrier to future radical prostatectomy when indicated.

The inclusion of two conference abstracts in this review adds valuable, albeit preliminary, evidence to this uncommon clinical scenario [22,23]. These reports complemented the full-text data by providing additional insight into early functional outcomes and comparisons with nonsurgical treatments. Nevertheless, the limitations inherent to abstract-only data—such as incomplete methodology and absence of peer review—should be acknowledged.

Overall, the current evidence base is constrained by several limitations. Most studies are retrospective and single-center with relatively small sample sizes and heterogeneous outcome reporting. The absence of standardized definitions for continence, potency, and complications further limits comparability and affects the generalizability of the conclusions. More specifically, some defined continence strictly as the use of no pads per day, while others accepted 0–1 pad/day, and assessment time points varied. Potency was assessed through different methods, including the SHIM questionnaire or subjective patient-reported function, while several studies did not define erectile recovery at all. Future research should prioritize prospective, multicenter investigations that employ uniform outcome measures and assess the impact of surgical experience, robotic technology, and reconstructive strategies on postoperative recovery. The potential role of advanced preoperative imaging and intraoperative navigation tools such as augmented reality guidance in optimizing dissection after HoLEP also warrants further exploration as studies have shown promising results [27,28,29].

In summary, radical prostatectomy after HoLEP, though technically demanding, is a safe and effective procedure that provides oncological control equivalent to primary prostatectomy while maintaining acceptable long-term functional outcomes. Awareness of altered post-enucleation anatomy, careful surgical planning, and performance by experienced surgeons are essential to achieving optimal results in this increasingly relevant patient population.

## 5. Clinical Implications

The findings of this review have several important implications for clinical practice. Radical prostatectomy after HoLEP should be recognized as a technically feasible and oncologically safe treatment option for patients with localized prostate cancer, provided that the procedure is performed by experienced surgeons in specialized centers. Preoperative counseling is essential to ensure that patients are fully informed about the potential for increased operative difficulty and the possibility of delayed early continence recovery compared with primary radical prostatectomy. Such counseling allows for appropriate expectation management and supports shared decision-making regarding treatment options resulting in less decision regret and better quality of life [30,31,32].

Given the anatomical alterations caused by HoLEP—particularly at the bladder neck and prostatic apex—these operations are best performed in high-volume robotic centers with expertise in complex pelvic surgery and reconstructive techniques. Surgeons familiar with post-enucleation anatomy and proficient in meticulous bladder-neck reconstruction are better equipped to minimize intraoperative challenges and optimize both oncological and functional outcomes.

From a broader perspective, the evidence synthesized in this review supports the continued use of HoLEP as a first-line surgical treatment for benign prostatic hyperplasia, even in patients who may later require radical prostatectomy. Prior HoLEP does not preclude the safe and effective performance of oncologic surgery, nor does it compromise cancer control. This reinforces HoLEP’s status as a durable and oncologically neutral intervention for bladder outlet obstruction, providing long-term functional benefits without jeopardizing future treatment options.

As the number of men undergoing HoLEP continues to rise, the subset who later develop prostate cancer will inevitably grow. Urologists should therefore remain familiar with the unique technical and anatomical considerations of post-HoLEP radical prostatectomy. Ongoing training, careful patient selection, and adoption of refined robotic techniques will be critical to maintaining the high standards of safety and efficacy demonstrated in recent studies.

## 6. Conclusions

Radical prostatectomy after HoLEP is feasible, safe, and oncologically sound when performed by experienced surgeons. Although prior HoLEP may increase technical complexity and slightly delay early continence recovery, long-term functional and oncological outcomes appear comparable to primary radical prostatectomy. These findings support HoLEP as an appropriate BPH treatment even for men who may later require definitive prostate cancer surgery. Further prospective multicenter studies with standardized outcome reporting are needed to better characterize predictors of recovery and refine surgical strategies in this growing patient population.

## Figures and Tables

**Table 1:** Characteristics of Studies on Radical Prostatectomy (RP) after Holmium Laser Enucleation of the Prostate (HoLEP).

Study (Year)	Country/Center	Design	N (Post-HoLEP RP)	Surgical Approach	Interval HoLEP–RP (Months)	Median Follow-Up (Months)	Key Outcomes Reported	Definition of Continence/Potency
Suardi et al., 2008 [16]	Italy	Retrospective comparative	15	Open (nerve-sparing RRP)	5.8 ± 5.3	23.8 ± 10.5	Feasible NS-RRP; PSM 6.7%; continence 93% at 6 mo; similar erectile recovery	Continence: 0 pads/day; Potency: erections sufficient for intercourse (with or without PDE5 inhibitors)
Gellhaus et al., 2015 [17]	USA	Retrospective matched	11	Robot-assisted	48 (1–156)	24	Feasible; more difficult bladder neck dissection; continence 27% vs. 64% controls; lower SHIM scores	Continence: 0 pads/day; Potency: SHIM score reported (lower in HoLEP group); no cut-off defined
Abedali et al., 2020 [18]	USA	Retrospective	32	Robot-assisted	24	30	Longer OT; slower continence recovery; similar PSM and BCR rates	Continence: 0 pads/day; Potency: not defined or reported
Kretschmer et al., 2021 [19]	Multicenter	Multi-institutional retrospective	95	Open or Robot-assisted	Not specified	50.5	PSM 14%; no impact on BCR-free survival; worse 1-yr continence (68% vs. 81%); similar erectile recovery	Continence: 0 pads/day; Potency: SHIM ≥ 17 considered potent; nerve-sparing reported
Verze et al., 2016 [20]	Italy	Retrospective single-surgeon	98 (various prior surgeries incl. HoLEP)	Laparoscopic	≥4	24	Longer OT, more EBL, higher anastomotic stricture; similar oncologic results; delayed continence	Continence: 0–1 pad/day; Potency: not clearly defined
Banno et al., 2023 [21]	Japan	Retrospective	20	Robot-assisted	20	24	Comparable OT, EBL; no major complications; similar long-term continence	Continence: 0 pads/day; Potency: erections sufficient for intercourse (self-reported)
Rosiello et al., 2020 [22]	Italy and Belgium	Retrospective, single-institution	7	Robot-assisted	41 (IQR 38–52)	25 (IQR 18–36)	No difference in OT, EBL, PSM, or BCR; continence 71% at 3 mo; feasible and safe	Continence: 0 pads/day; Potency: not assessed or reported
Piroozi et al., 2023 [23]	USA	Retrospective	33	Robot-assisted	Not stated	>12	Similar AUA/QoL to radiation therapy; higher incontinence (73% vs. 28%) after RP	Continence: AUA Symptom Score and pad use (0–1 pad/day); Potency: not assessed or reported

RRP = radical retropubic prostatectomy; NS-RRP = nerve-sparing radical retropubic prostatectomy; OT = operative time; EBL = estimated blood loss; PSM = positive surgical margin; BCR = biochemical recurrence; SHIM = Sexual Health Inventory for Men; AUA = American Urological Association; QoL = quality of life; IQR = interquartile range; NA = not available.

**Table 2:** Summary of Perioperative, Oncologic, and Functional Outcomes after RP following HoLEP.

Outcome Category	Parameter	Range/Representative Values	Main Findings Across Studies
Perioperative	Operative time	+20–60 min longer than primary RP	Increased due to fibrosis and distorted anatomy [17,18,20]
Perioperative	Estimated blood loss	+50–100 mL	Mildly higher, not clinically significant [20,21]
Perioperative	Major complications (≥Clavien III)	<5%	Rare; similar to controls [19,22]
Perioperative	Bladder neck reconstruction	20–50% cases	Common due to tissue fibrosis [17,20]
Oncologic	Positive surgical margin rate	6–20%	Comparable to primary RP [16,19,21]
Oncologic	Biochemical recurrence	0–26% (crude rates)	Cumulative Biochemical Recurrence-Free Survival: no difference vs. controls [19,22]
Oncologic	Cancer-specific survival	100% (all series)	No deaths during follow-up
Functional	Continence at 12 months	65–90%	Slower early recovery, long-term outcomes comparable [16,19,22]
Functional	Erectile function recovery	Variable (30–60%)	Feasible when nerve-sparing possible [16,19]
Functional	Incontinence vs. radiation	73% vs. 28%	Higher after RP [23]

## Data Availability

Data sharing is not applicable.
